# Questions concerning tyrosine kinase-inhibitor therapy and transplants in chronic phase chronic myeloid leukaemia

**DOI:** 10.1038/s41375-022-01522-3

**Published:** 2022-03-25

**Authors:** Michele Baccarani, Francesca Bonifazi, Simona Soverini, Fausto Castagnetti, Gabriele Gugliotta, Wael Saber, Noel Estrada-Merly, Gianantonio Rosti, Robert Peter Gale

**Affiliations:** 1 IRCCS Azienda Ospedaliero –Universitaria di Bologna, Bologna, Italy; 2 Department of Experimental, Diagnostic and Specialty Medicine (DIMES), Hematology ‘Lorenzo e Ariosto Seràgnoli’, University of Bologna, Bologna, Italy; 3 Center for International Blood and Marrow Transplant Research), Milwaukee, WI USA; 4 Division of Hematology/Oncology, Department of Medicine, Medical College of Wisconsin, Milwaukee, WI USA; 5 IRCCS/IRST “Dino Amadori”, Meldola, FC Italy; 6 Haematology Research Centre, Department of Immunology and Inflammation, Imperial College London, London, UK

**Keywords:** Medical research, Haematological diseases

## Abstract

In this provocative commentary, we consider several questions posed by the late chronic myeloid leukaemia (CML) expert Prof. Michele Baccarani, which he challenged us to address after his death. He noted only a small proportion of people with chronic phase CML receiving tyrosine kinase-inhibitor (TKI)-therapy are likely to achieve sustained therapy-free remission (TFR) and even fewer are likely to be cured. Persons most likely to fail TKItherapy can be identified at diagnosis or soon after starting TKI-therapy. These persons are likely to need lifetime TKI-therapy with attendant risks of adverse events, cost and psychological consequences. Allogeneic transplants achieve much higher rates of leukaemia-free survival compared with TKI-therapy but are associated with transplant-related adverse events including an almost 20 percent risk of transplant-related deaths within 1 year post-transplant and a compromised quality-of-life because of complications such as chronic graft-versus-host disease. Subject-, disease- and transplant-related co-variates associated with transplant outcomes are known with reasonable accuracy. Not everyone likely to fail TKI-therapy is a transplant candidate. However, in those who candidates are physicians and patients need to weigh benefits and risks of TKI-therapy versus a transplant. We suggest transplants should be more often considered in the metric when counseling people with chronic phase CML unlikely to achieve TFR with TKI-therapy. We question whether we are discounting a possible important therapy intervention; we think so.

Scientists who fall deeply in love with their hypothesis are proportionately unwilling to take no as an experimental answer.

Sir Peter Medawar

## Introduction

Before the development of imatinib and other tyrosine kinase-inhibitors (TKIs) allogeneic haematopoietic cells transplants were a common intervention in chronic phase chronic myeloid leukaemia (CML) in appropriate persons and were the only approach to cure. With the remarkable success of TKI-therapy transplants for chronic phase CML became rare with less than 300 reported to the Centre for International Blood and Marrow Research (CIBMTR) in 2014–2016. However, it’s become clear that despite excellent survivals with TKI-therapy in many but not all countries only a small proportion of people are likely to achieve therapy-free remission (TFR) and even fewer cured. There is also considerable debate over the most appropriate target of TKI-therapy. Should it be population-adjusted survival, TFR or cure? When population-adjusted survival is the target transplants are unlikely to be better than TKI-therapy in most, but not all persons such as those failing to respond to TKI-therapy and those with some *ABL1* mutations, high-risk additional cytogenetic abnormalities (ACAs) and/or with other signs of leukaemia progression. Also, when the goal of TKI-therapy is TFR or cure transplants may be appropriate for some persons. In this Perspective, we present 10 questions for future research on the roles of TKI-therapy and transplants in chronic phase CML, questions raised by the late CML expert Prof. Michele Baccarani.

## What is the appropriate goal of CML therapy?

The optimal goal of CML therapy is cure resulting in normal sex- and age-adjusted survival with a normal *quality-of-life* (QoL) [1–3]. Unfortunately, cure is achieved in few people with CML [4, 5]. An intermediate goal is achieving near normal age- and sex-matched adjusted survival off tyrosine kinase inhibitor (TKIs)-therapy referred to as therapy-free remission (TFR) [2–10].

## Are TKI therapy goals changing and which TKI is *best* to achieve which goal?

Several TKIs are commercially available to treat CML in many but not all countries and at considerably different costs [11]. Imatinib, nilotinib, dasatinib, bosutinib and, in Korea, radotinib are approved for initial therapy, and ponatinib and asciminib in the US for 2^nd^ and 3^rd^-line therapies [2, 7–9, 12–14]. Imatinib is less potent and does not inhibit several *BCR::ABL1* mutations many of which are sensitive to the other TKIs, except *BCR::ABL1*^T315I^ which is inhibited only by ponatinib and asciminib [14–16]. All TKIs cause adverse events, with some clinically relevant differences particularly for cardio-vascular and pulmonary complications. Imatinib is the safest [17]. Safety profiles of TKIs are considered largely manageable with favourable *benefit-to-risk* ratios. Cost and compliance are also important considerations and often influence TKI choice, especially in resource-poor geospaces [6, 17–21].

The therapeutic strategy for CML when imatinib was the only approved TKI was simple. After nilotinib and dasatinib were approved for initial therapy and bosutinib and ponatinib for subsequent therapy, several different strategies were developed, followed by debate and competition [22–32]. This competition is mainly over which TKI is the *best* initial therapy in the context of faster, deeper molecular responses obtainable with 2nd-generation TKIs (2G-TKIs; nilotinib, dasatinib, bosutinib) and over the switch from imatinib to 2G-TKIs if there is a sub-optimal response to imatinib [2, 6–9, 19]. This is important because molecular response, particularly major molecular response (MMR; *BCR::ABL1* ≤ 0.1% on the International Scale) is widely considered the best surrogate for survival [31, 32]. However, there are no convincing data supporting the initial use of a 2G-TKI being associated with better progression-free survival (PFS), probability of achieving TFR or survival [2, 7–9, 23]. Consequently, whether the advantage of 2G-TKIs over imatinib in achieving faster and deeper molecular responses translates into a higher rate of TFR and *operational* cures remains unproven and can only be tested in a randomized controlled trial [2, 7–9, 21]. Such a trial is unlikely to be done.

## Are current recommendations for TKI-therapy appropriate?

CML therapy recommendations are continuously modified with success inviting to more ambitious goals. Five-year survival of persons with CML is now 80–90% in the European geospaces, with about one-half of deaths occurring from unrelated causes [1, 33]. Survival is like that of sex- and age-adjusted people without CML in Europe, but not in the US and certainly not in resource-poor countries [33]. Considerable data suggest people with CML achieving a stable deep molecular response (DMR; ≥MR^4^; 4-log *BCR::ABL1* transcript decrease from the standardized baseline, corresponding to a transcript level ≤0.01% on the International Scale) can discontinue therapy, about one-half of whom achieving TFR [34–49]. The clinical advantage of TFR over lifelong TKI-therapy is obvious, but the road to achieving this goal is not simple, cheap or rewarding for everyone. Some people choose not to stop TKI therapy for diverse reasons, usually fear of leukaemia recurrence [50].

There is controversy on how to best use TKIs. Which are the best and most cost-effective strategies to achieve TFR, to optimize survival and improve QoL [2, 7–9, 35, 36, 38, 41–46]? Which strategy(ies) should be used when someone does not meet proposed TKI stopping criteria or fails because of molecular, cytogenetic and/or haematologic leukaemia recurrence? How can we limit adverse events (AEs) associated with lifelong TKI-therapy and complications of more intensive therapies aimed at achieving TFR? Put otherwise, the main issues are: which TKI, at what dose and for how long, alone or with other drugs? But there is another important consideration. TKI-therapy rarely cures CML, as we discussed elsewhere [5]. If so, should the only therapy of CML be TKIs?

## How quickly is deep molecular response achieved with TKI-therapy?

Achieving a stable DMR (*BCR::ABL1* ≤ 0.01% on the International Scale) is widely considered to be necessary before stopping TKI-therapy [2, 7–9, 22, 26, 27, 34–37, 43–46]. DMR rates in 30 cohorts of newly-diagnosed subjects receiving different TKIs at different doses, alone or with other drugs such as interferon-alfa (IFNα) or low-dose cytarabine, are displayed in Table 1. These rates, typically reported as probability of achieving a DMR within a specified interval rather than as proportion of subjects achieving a DMR, over-estimate the proportion of subjects eligible to discontinue TKI therapy. These studies report rates of 20–70% with imatinib-based regimes and 60–80% with 2G-TKIs. Comparably, 5-year rates of MR^4.5^ are 5–35% and 35–70% (Table 2). Interestingly, although achieving MR^4^ is universally considered as a critical target, reported DMR rates vary widely with the same therapy and despite of standardization of real-time quantitative polymerase chain reaction (RT-qPCR), used for response assessment.

**Table 1:** MR^4^ response rates (percentage) by or at 3-, 5- and 10-years from initial TKI therapy. Only studies with ≥ 3-year follow-up are displayed. All rates are ‘by’ except for those reported for Guilhot et al. [27].

Ref.	Study	Initial TKI	*N*	Median age (y)	3-y MR^4^ rate	5-y MR^4^ rate	10-y MR^4^ rate
De Lavallade et al. [88]	Hammersmith	IM 400	204	46	15	20	NR
Castagnetti et al. [89]	GIMEMA	IM 400/800	559	52	25^a^	61^b^	NR
O’Brien et al. [29]	UK SPIRIT 2	IM 400	407	53	NR	57	NA
Hochhaus et al. [24], Kantarjian et al. [26]	ENESTnd	IM 400	283	46	26	42	50
Zhang et al. [69]	Peking	IM 400	1379	40	NR	NR	54^c^
Guilhot et al. [27]	French SPIRIT	IM 400	223	50	36	37	40
Hehlmann et al. [30, 31]	German Study IV	IM 400	400	53	49	66	81
Guilhot et al. [27]	French SPIRIT	IM 400 + LDAC	172	51	35	41	48
Hehlmann et al. [30, 31]	German Study IV	IM 400 + LDAC	158	51	49	68	86
Guilhot et al. [27]	French SPIRIT	IM 400 + IFN_α_	221	55	44	48	40
Hehlmann et al. [30, 31]	German Study IV	IM 400 + IFN_α_	430	53	51	67	83
Guilhot et al. [27]	French SPIRIT	IM 600	171	51	36	49	50
Hehlmann et al. [30, 31]	German Study IV	IM 800	420	51	59	69	81
Geelen et al [90]	Dutch	IM 400, NIL 600, DAS 100	434	58	41^a^	69^d^	NA
O’Brien et al. [29]	UK SPIRIT 2	DAS 100	407	52	NR	78	NA
Hochhaus et al. [24], Kantarjian et al. [26]	ENESTnd	NIL 600	282	47	50	66	70
Gugliotta et al. [91]	GIMEMA	NIL 600/800	472	52	76	NR	NA
Gugliotta et al. [92]	GIMEMA	NIL800	73	51	70	76	83
Hochhaus et al. [24], Kantarjian et al. [26]	ENESTnd	NIL 800	281	47	44	63	68
Masarova et al. [93]	MDACC	NIL 800	122	51	66	73	82

TKI doses are in mg/d. Percentages are rounded.

*MR*^*4*^ *BCR::ABL1* ≤ 0.01%^IS^, *IM* imatinib, *NIL* nilotinib, *DAS* dasatinib, *IFN*_*α*_ interferon-α, *LDAC* low dose cytarabine, *NR* not reported, *NA* not available, *GIMEMA* Gruppo Italiano Malattie Ematologiche dell’Adulto, *JALSG* Japan Adult Leukemia Study Group, *MDACC* MD Anderson Cancer Center.

^a^2-y, ^b^6-y, ^c^7-y, ^d^4-y.

**Table 2:** MR^4.5^ response rates (percentage) by or at 3-, 5- and 10-years. Only studies with ≥3 years follow-up. All rates are ‘by’ except for those reported for Guilhot et al. [27].

Ref.	Study	Initial TKI	*N*	Median age (y)	3-y MR^4.5^ rate	5-y MR^4.5^ rate	10-y MR^4.5^ rate
De Lavallade et al. [88]	Hammersmith	IM 400	204	46	4^f^	8^f^	NR
Branford et al. [22]	Adelaide	IM 400/600/800	423	NR	NR	NR	52^a^
Cortes et al. [25]	DASISION	IM 400	260	49	13	33	NA
Hochhaus et al. [24], Kantarjian et al. [26]	ENESTnd	IM 400	283	46	15	31	39
Zhang et al. [69]	Peking	IM 400	1373	41	NR	NR	43^b^
Guilhot et al. [27]	French SPIRIT	IM 400	223	50	24	23	27
Hehlmann et al. [30, 31]	German Study IV	IM 400	401	53	35	49	67
Guilhot et al. [27]	French SPIRIT	IM 400 + LDAC	172	51	22	22	34
Hehlmann et al. [30, 31]	German Study IV	IM 400 + LDAC	158	51	31	50	70
Guilhot et al. [27]	French SPIRIT	IM 400 + IFN_α_	221	55	26	33	29
Hehlmann et al. [30, 31]	German Study IV	IM 400 + IFN_α_	430	53	38	54	74
Guilhot et al. [27]	French SPIRIT	IM 600	171	51	24	31	36
Hehlmann et al. [30, 31]	German Study IV	IM 800	399	52	43	58	71
Etienne et al. [43]	French	IM 400, DAS 100,NIL 600	398	62	31^e^	40^e^	52^e^
Geelen et al. [90]	Dutch	IM 400, DAS 100, NIL 600	434	58	30^c^	56^d^	57
Cortes et al. [25]	DASISION	DAS 100	259	46	20	42	NA
Matsumura et al. [28]	JALSG	DAS 100	227	53	45	NA	NA
Matsumura et al. [28]	JALSG	NIL 600	227	53	41	NA	NA
Hochhaus et al. [24], Kantarjian et al. [26]	ENESTnd	NIL 600	282	47	32	54	61
Hochhaus et al. [24], Kantarjian et al. [26]	ENESTnd	NIL 800	281	47	28	52	61
Masarova et al. [93]	MDACC	NIL 800	122	51	61	72	75

TKI doses are in mg/d. Percentages are rounded.

*MR*^*4.5*^ *BCR::ABL1* ≤ 0.0032%^IS^, other abbreviations as in Table 1.

^a^8-y, ^b^7-y, ^c^2-y, ^d^4-y, ^e^sustained (at least 24 months), ^f^rates of ‘complete molecular response (CMR)’ defined as two consecutive samples with no detectable transcripts.

## How many people can successfully discontinue TKI therapy?

Expert consensus statements and clinical practice guidelines recommend >5 years of imatinib and >3 to 5 years of a 2G-TKI, with a response ≥ MR^4^ for ≥2 years [2, 7–9, 38, 44, 46, 48, 49]. Convincing data supporting these recommendations are lacking [10]. If applying these criteria, only about 45% of people receiving imatinib might achieve MR^4^ at 3 years. Assuming they remain in MR^4^ for other 2 years it can be estimated that about 45% would become eligible to stop TKI therapy at ≥ 5 years. In persons receiving 2G-TKIs alone or with other drugs, this estimate is only slightly higher, about 50%. Combining these data only 10–25% of people will be eligible to stop TKI-therapy, which can be estimated to be successful in about one-half of people or about 10% of everyone with chronic phase CML (see below).

Many studies have reported the rate of TFR on > 2000 subjects cumulatively, but the real rate of successful TKI-stopping in persons with newly-diagnosed chronic phase CML is rarely reported. We estimate this proportion in Table 3 along with the proportion still in TFR at last contact at only 10–25%.

**Table 3:** Percentage of newly-diagnosed CML patients meeting TKI discontinuation criteria and achieving stable TFR. Discontinuation and TFR criteria are arbitrarily defined, differ between studies and are often not pre-specified. Data are from retrospective analyses.

Ref	Study	Initial TKI	*N*	Median follow-up (y)	Met discontinuation criteria	Discontinued	Achieved Stable TFR
Branford et al. [22]	Adelaide	IM 400/600/800	423	8	37%	NR	NR
Geelen et al. [90]	Dutch	IM 400 (75%), 2GTKIs (25%)	382	10	31%	10%	NR
Flygt et al. [48]	Swedish	Mainly IM 400	548	9	NR	23%	12%
Etienne et al. [43]	French	Mainly IM 400	398	7	10%–55%	46%	12%
Kantarjian et al. [26]	ENESTnd	IM 400	283	10	30%	NR	NR
Guilhot et al. [27]	French SPIRIT	IM 400 + LDAC or + IFN_α_ or IM 600	787	13.5	NR	44%	18%
Gugliotta et al. [92]	GIMEMA	NIL 800	73	10	NR	33%	25%
Kantarjian et al. [26]	ENESTnd	NIL 600	282	10	49%	NR	NR
Kantarjian et al. [26]	ENESTnd	NIL 800	281	10	47%	NR	NR

TKI doses are in mg/d. Percentages are rounded.

Abbreviations as in Table 1. 2GTKIs: second-generation TKIs.

## Are survival results of TKI-therapy adequate?

Survival from diagnosis is the most reliable study endpoint because it requires no further definition and *time-to-event* data are evaluable in almost all subjects. In contrast, definitions of other endpoints such as failure-free survival (FFS), progression-free survival (PFS) and CML-related survival differ between studies. For example, identifying the cause(s) of death may be subjective and difficult to accurately ascertain in retrospective analyses. Survival data of newly-diagnosed people initially treated with TKIs are reasonably consistent with 1- and 2-year survivals of >95% and 3-, 5- and 10-year survivals >80% in persons receiving imatinib or a 2G-TKI as initial therapy (Table 4).

**Table 4:** Survival of subjects receiving TKI therapy.

Ref.	Study	Initial TKI	*N*	Median age (y)	3-y (%)	5-y (%)	10-y (%)
Castagnetti et al. [94]	EUTOS	IM 400	236	60	93	85	NA
Castagnetti et al. [89]	GIMEMA	IM 400/800	559	52	NR	89^b^	NA
de Lavallade et al. [88]	Hammersmith	IM 400	204	46	96	83	NA
Hochhaus et al. [32]	IRIS	IM 400	553	50	92	89	83
Guilhot et al. [27]	French SPIRIT	IM 400	223	50	95	95	90
O’Brien et al. [29]	UK SPIRIT 2	IM 400	407	53	NR	91	NR
Hehlmann et al. [30, 31]	German Study IV	IM 400	400	53	96	88	80
Hochhaus et al. [24], Kantarjian et al. [26]	ENESTnd	IM 400	283	46	94	92	88
Zhang et al. [69]	Peking	IM 83%, 2G-TKI 17%	1373	40	NR	94^c^	NA
Cortes et al. [25]	DASISION	IM 400	260	49	95^d^	90	NR
Guilhot et al. [27]	French SPIRIT	IM 400 + LDAC	172	55	95	91	85
Hehlmann et al. [30, 31]	German Study IV	IM 400 + LDAC	158	51	NR	86	84
Hehlmann et al. [30, 31]	German Study IV	IM 400 + IFN_α_	430	53	95	88	84
Guilhot et al. [27]	French SPIRIT	IM 400+ IFN_α_	221	51	95	95	89
Kalmanti et al. [95]	German Study IV	IM 400 ± LDAC or + IFN_α_ or IM 800	120	16–29^a^	NR	97	NR
Kalmanti et al. [95]	German Study IV	IM 400 ± LDAC or + IFN_α_ or IM 800	383	30–44^a^	NR	94	NR
Pfirrmann et al [96]	EUTOS	IM 400 > 80%	2290	51	NR	NR	89^e^
Geelen et al. [90]	Dutch	IM 77%, 2G-TKI 23%	382	58	92^d^	85^f^	NA
Etienne et al. [43]	French	IM 73%, 2G-TKI 27%	398	64 IM, 54 2G-TKI	NR	90	81
Jain et al. [23]	MDACC	IM 57%, NIL 21%, DAS 21%	197	14–44^a^	98	96	87
O’Brien et al. [29]	UK SPIRIT 2	DAS 100	407	53	NR	92	NA
Matsumura et al. [28]	JALSG	DAS 100	227	53	99	NA	NA
Cortes et al. [25]	DASISION	DAS 100	259	46	95^d^	91	NA
Hochhaus et al. [24], Kantarjian et al. [26]	ENESTnd	NIL 600	282	47	95	94	88
Matsumura et al. [28]	JALSG	NIL 600	227	53	99	NA	NA
Hochhaus et al. [24], Kantarjian et al. [26]	ENESTnd	NIL 800	281	47	97	96	95
Masarova et al. [93]	MDACC	NIL 800	122	51	97	93	88
Gugliotta et al. [92]	GIMEMA	NIL 800	73	51	97	96	95

TKI dose in mg/d. Percentages rounded.

Some data are estimated from graphs (±1%). Abbreviations as in Table 1.

^a^Age intervals instead of median, ^b^6-y, ^c^7-y, ^d^2-y, ^e^8-y, ^f^4-y.

## What are results of allogeneic haematopoietic cell transplants and have they improved?

**Fig. 1:**
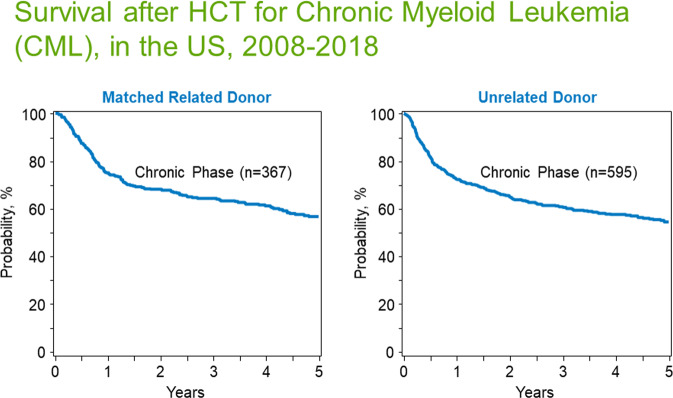
Survival after allogeneic transplants (2008–18) for chronic myeloid leukemia in chronic phase (from Phelan, R., Arora, M., Chen, M. Current use and outcome of hematopoietic stem cell transplantation: CIBMTR US summary slides, 2020). Left panel shows overall survival of patients with chronic myeloid leukemia in chronic phase transplanted from a matched related donor; right panel shows overall survival after allogeneic transplants from an unrelated donor. *n* number of patients transplanted.

CML transplants, once the most common transplant indication, are now uncommon. In 2020, <200 of >10,000 allotransplants reported to the CIBMTR were for CML, done mostly in persons in accelerated or blast phases. Outcomes from several transplant centres and registries of transplant outcomes in persons with chronic phase CML mostly done before 2012 are displayed in Table 5. A 5-year survival, not leukaemia-free survival (LFS), of about 60% is reported by the CIBMTR in 1,445 subjects with CML in chronic phase receiving transplants from HLA-identical siblings (Fig. 1). Goldman *et al*. [51] reported data from 2,221 persons in chronic phase CML receiving transplants from HLA-identical siblings (*N* = 1,692) or HLA-matched unrelated donors (*N* = 639) alive and leukaemia-free at 5 years posttransplant. Ten- and 15-year posttransplant LFS were 91% (95% Confidence Interval [CI], 90, 92%) and 83% (81, 85%). Comparable cumulative incidences of relapse (CIR) were 4% (3, 5%) and 7% (5, 8%). There was a slow but steady relapse risk after 5 years posttransplant with the latest relapse at 18 years. These data indicate a high cure in persons alive and without relapse at 5 years posttransplant.

**Table 5:** Survival after an allotransplant for CML in 1st chronic phase.

		Interval	*N*	Median age (y)	Conditioning	Donor	1-y	2-y	3-y	5-y	10-y
Millot et al. [97]	SGFMTC	1982–1998	42	14	MA	REL	87%	85%	77%	73%	73%
Cwynarski et al. [98]	EBMT	1985–2001	156	14	NR	REL	78%	75%	75%	72%	70%
Arora et al. [99]	CIBMTR	1988–2003	3514	36	MA	REL	74%	65%	63%	63%	60%
Arora et al. [99]	CIBMTR	1988–2003	531	37	MA	UNR	70%	63%	58%	55%	50%
Radich et al. [100]	Seattle^a^	1995–2000	131	43	MA	REL	91%	86%	86%	NA	NA
Gratwohl et al. [61]	German Study III^a^	1997–2004	151	38	MA	REL	90%	85%	82%	78%	76%
Gratwohl et al. [61]	German Study III	1997–2004	148	41	MA	UNR	97%	85%	77%	76%	76%
Bacher et al. [62]	German Registry	1998–2004	1084	40	MA 62%	REL 61%	67%	65%	65%	64%	64%
Ohashi et al. [101]	Japanese Registry	2000–2009	531	40	MA 89%	UNR 51%	87%	86%	85%	85%	78%
Chaudury et al. [102]	CIBMTR	2001–2010	224	24	MA	REL	90%	88%	85%	83%	NA
Chaudury et al. [102]	CIBMTR	2001–2010	225	24	MA	UNR	80%	76%	72%	68%	NA
Lee et al. [103]	Korean^a^	2001–2012	47	32	MA 77%	UNR 43%	88%	86%	86%	NA	NA
Lee et al. [103]	Korean	2001–2012	50	33	MA 48%	UNR 42%	90%	86%	80%	NA	NA
Koenecke et al. [104]	EBMT	2002–2005	193	31	MA	REL	90%	87%	86%	85%	84%
Saussele et al. [105]	German Study IV^a^	2003–2008	19	35	MA 79%	REL 53%	95%	88%	88%	NA	NA
Saussele et al. [105]	German Study IV	2003–2008	37	38	MA 65%	UNR 70%	95%	95%	94%	NA	NA

^a^Data are estimated from graphs (±1%). *SGFMTC* Société Française de Greffe de Moelle et de Thérapie Cellulaire, *EBMT* European Group for Marrow and Blood Transplantation, *CIBMTR* Center for International Blood and Marrow Transplantation, *MA* myelo-ablative, *REL* related donor, *UNR* unrelated donor, NR not reported.

Because these data are predominately from the pre-TKI era, we analyzed CIBMTR data from the 238 transplants done between 2014 and 2016 in persons with CML in 1st chronic phase from all donors. One-year non-relapse mortality (NRM) was 17% (12, 23%). Five-year CIR was 18% (13, 23%) with almost all relapses with the 1st year posttransplant. 5-year survival was 68% (61, 74%). These outcomes very likely reflect strong selection biases operating in both directions. First, persons responding poorly to TKI-therapy are more likely to receive a transplant than good responders. In contrast, transplants were likely done in young persons with a good performance score, well-matched donors and few co-morbidities. Consequently, these summary outcomes data should be viewed cautiously.

There are several recent transplant advances including: (1) a donor such as an HLA-haplotype-matched relative for almost everyone; (2) increasing use of blood cells over bone marrow grafts; (3) development of less intensive pretransplant conditioning regimens (termed *reduced-intensity* condition [RIC]) applicable to older persons; (4) use of posttransplant cyclophosphamide as well as anti-lymphocyte globulin (ATG/ATLG) reducing risks of acute and chronic graft-*versus*-host disease (G*v*HD) seemingly without increasing relapse risk (although this has not been critically tested in CML) [52–54]; (5) better supportive care; and others. These advances have decreased transplant-related deaths by about 20% and increased survival by about 10% [55]. Whether these advances apply to transplants done for chronic phase CML is unknown.

As indicated, leukaemia recurrence is uncommon after allotransplants for chronic phase CML [51, 55–57]. Much of this anti-leukaemia efficacy results from an allogeneic effect [54, 58]. Transplants from genetically-identical twins, T-cell-depleted grafts and transplants in persons without G*v*HD have substantially higher CIRs, reflecting immune-mediated anti-leukaemia effect. Early relapses are often successfully treated by stopping posttransplant immune suppression, giving donor lymphocyte infusions (DLIs) and/or giving TKIs [59]. Late relapses are rare, but relapse risk continues indefinitely [51]. Allotransplants done in chronic phase result in about 80% 15-year LFS [51]. However, some persons develop chronic G*v*HD or other complications which compromise QoL and are sometimes fatal. Other considerations which are incompletely resolved are the impact of pre- and posttransplant TKI-therapy on transplant outcomes.

## Who should be considered for a transplant in chronic phase?

The question of who should receive a transplant in chronic phase is complex and controversial. Probably the clearest indication is in drug compliant persons failing to respond to TKI-therapy and those with some *BCR::ABL1* mutations, high-risk additional cytogenetic abnormalities (ACAs) and/or with other signs of leukaemia progression [60]. There are persons who cannot tolerate TKI therapy, or who develop severe adverse events which cannot be managed by dose adjustment of switching to a different TKI. They are a minimal part of patients. But they are.

A more complicated question is whether a transplant is an appropriate option in a person likely to have good survival but unlikely to achieve TFR and who therefore require lifelong TKI-therapy. The first issue is whether such persons can be accurately identified and when. Several predictive models have been developed which predict failure of TKI-therapy but none has a Concordance (C)-statistic >0.80. The next issue is whether it’s possible to accurately predict transplant outcomes. Again, several predictive models have been developed with similar C-statistics. A third issue is suitability of someone to receive a transplant including age, co-morbidities, donor availability and fiscal resources.

There cannot be an uniform correct answer. For example, a younger person is more likely to accept the immediate survival disadvantage of transplants for a substantial probability of cure whereas an older person may not. Another consideration is a personal satisfaction/dis-ratification with remaining on lifelong TKI-therapy. There are also fiscal considerations. In some resource-poor geospaces there may be a substantial cost saving to receiving a transplant. And one should not ignore the important impact of patient and physician risk-taking attitude which we discuss below.

## Aren’t most people with CML too old to receive a transplant?

Most studies of CML therapy including transplants are in resource-rich geospaces where median age at diagnosis is about 60 years [61–64]. However, in some Asian and African countries median age at diagnosis is <50 years [65–67]. In an international review of >40,000 subjects with newly diagnosed CML, the rate of adults <50 years old in Asia and Africa was about 70% compared with 35% in Europe, increasing the proportion of persons with CML who might be considered for a transplant [64]. Transplant studies are obviously skewed towards younger persons.

## Do we need to reconsider use of transplant in chronic phase CML?

Despite recent progress, few persons with chronic phase CML receiving TKI therapy achieve TFR, and even fewer, if any, are cured [68]. Most persons failing to achieve arbitrarily specified TKI-therapy response goals can be reasonably accurately identified at diagnosis or soon after starting TKI therapy [69]. Rates of remaining leukaemia-free are certainly lower and cure rates higher in persons receiving a transplant. However, there are important *caveats* when interpreting these data: (1) few transplants have been done for CML recently, limiting the certainty of estimating outcomes; (2) there are subject selection biases favouring transplants including younger age, better performance score and fewer co-morbidities in transplant recipients compared with persons receiving TKIs. For example, median age of the CIBMTR cohort we describe above is 46 years, substantially younger than the median age of persons with CML of predominately European descent; (3) selection biases against transplant recipients who are more likely to have had a worse prognosis at diagnosis or soon thereafter compared with those receiving only TKI therapy; and (4) the almost 20% 1-year mortality associated with transplants and risk of transplant-related complications such as chronic G*v*HD.

At diagnosis, most physicians and persons with chronic phase CML are understandably reluctant to accept a 1-year TRM of almost 20% without a trial of TKI-therapy to determine whether the person is amongst the small proportion of those likely to achieve TFR and possibly cure. However, there are several time-dependent predictive and prognostic models and scores which enable physicians to estimate the likelihood of success of TKI therapy in achieving TFR reasonably early after starting TKI therapy. At this point, in persons who are potential transplant candidates, physicians and patients must choose between probable lifetime TKI therapy with attendant medical, physical and psychological costs versus likelihood of success and risks of a transplant [11, 70–73]. On the TKI therapy side of the calculus are considerations such as estimating the likelihood of adverse events, costs and risk tolerance. On the transplants side of the calculus are co-variates correlated with outcomes such as age, co-morbidities, donor HLA-matching, graft-type, pretransplant conditioning and posttransplant immune suppression regimens and others [74–76]. Of note, subject-, disease- and transplant-related predictive and prognostic co-variates previously operating in persons receiving and possibly failing TKI-therapy need confirmation.

A critical comparison of LFS or survival between TKI-therapy and transplants in comparable persons can only come from randomized controlled trials. Such a trial has not and will not be done. Also, the issue is not whether one or the other therapy is *better* but which therapy is more appropriate for different persons at different times after CML diagnosis and after observing response to TKI therapy [22, 23, 26, 27, 43, 44, 49, 77]. Both therapies have worse outcomes in older people, people with a poor performance score and those with co-morbidities, but these gradients are steeper for transplant recipients compared with persons receiving TKIs. Also older persons receiving TKI therapy are less likely to be therapy compliant, achieve TFR and remain on lifelong TKI therapy with attendant impacts on QoL. This is especially true for 2G-TKIs [2, 5–8].

A transplant is an increasingly relevant consideration in persons with a non-optimal response to TKI-therapy. Many of these persons can be identified by cytogenetic and molecular analyses, especially those with high-risk additional chromosome abnormalities (ACAs) and/or a 2nd *BCR::ABL1* or mutations in *TP53* and/or epigenetic modifier genes [78–84].

When the *best* therapy is controversial, physicians often rely on expert consensus statements and clinical practice guidelines. We discussed limitations of these tools elsewhere [85, 86]. However, our point is that panellists should consider adding transplants in persons with chronic phase CML during their deliberations [77, 87].

## Conclusion

This Perspective is a series of questions awaiting answers. They reflect questions Prof. Baccarani after a lifetime of CML research thought needed to be answered by the next generation of physicians interested in CML. Some of these questions can be answered by appropriately designed clinical trials. Others could theoretically be answered in clinical trials but for diverse reason such trials will not or cannot be done. Lastly, there are questions to which there is no one answer and certainly not one correct answer.

Medicine is an art, not a science. As the distinguished English, Canadian, American physician and medical educator Sir William Osler noted: *Medicine is a science of uncertainty and an art of probability* – Prof. Baccarani practiced a perfect blend of the science and art of medicine, of balancing uncertainty and probability. More mistakes are made by those who think they know the answer compared with those admitting uncertainty. Prof. Baccarani leaves us with these questions and challenges us to provide answers or at least to try. He was never afraid to challenge dogma or challenge answers to questions others thought answered. As Thomas Paine said: *He who dares not to offend cannot be honest*. *Omnia munda mundis*.

## Data Availability

CIBMTR supports accessibility of research in accord with the National Institutes of Health (NIH) Data Sharing Policy and the National Cancer Institute (NCI) Cancer Moonshot Public Access and Data Sharing Policy. The CIBMTR only releases de-identified datasets that comply with all relevant global regulations regarding privacy and confidentiality.
